# Supplementary material for: Empathic pain evoked by sensory and emotional-communicative cues share common and process-specific neural representations
Source: eLife. 2020 Sep 7;9:e56929. doi: 10.7554/eLife.56929 (PMC7505665; doi:10.7554/eLife.56929)
Supplement: Supplementary file 1. — NS vicarious pain, observation of noxious stimulation of body limbs induced vicarious pain; FE vicarious pain, observation of facial expressions of pain induced vicarious pain; NS control stimuli depict body limbs in similar but innocuous situations, FE control stimuli show neutral facial expressions. [file elife-56929-supp1.docx]

Post-fMRI subjective ratings for vicarious pain evoking stimuli (Mean ± SD).

|  | Categories of stimuli | | | |
| --- | --- | --- | --- | --- |
| Ratings | FE control | NS control | FE vicarious pain | NS vicarious pain |
| Pain intensity | 5.09 ± 7.34 | 8.49 ± 9.68 | 53.64 ± 21.43 | 71.43 ± 17.18 |
| Arousal | 16.79 ± 18.72 | 22.23 ± 18.74 | 54.48 ± 20.13 | 72.57 ± 17.53 |

NS vicarious pain, observation of noxious stimulation of body limbs induced vicarious pain; FE vicarious pain, observation of facial expressions of pain induced vicarious pain; NS control stimuli depict body limbs in similar but innocuous situations, FE control stimuli show neutral facial expressions.
