# Supplementary material for: Empathic pain evoked by sensory and emotional-communicative cues share common and process-specific neural representations
Source: eLife. 2020 Sep 7;9:e56929. doi: 10.7554/eLife.56929 (PMC7505665; doi:10.7554/eLife.56929)
Supplement: Transparent reporting form [file elife-56929-transrepform.docx]

***eLife’s* transparent reporting form**

We encourage authors to provide detailed information *within their submission* to facilitate the interpretation and replication of experiments. Authors can upload supporting documentation to indicate the use of appropriate reporting guidelines for health-related research (see [EQUATOR Network](http://www.equator-network.org/%20)), life science research (see the [BioSharing Information Resource](https://biosharing.org/)), or the [ARRIVE guidelines](http://www.plosbiology.org/article/info:doi/10.1371/journal.pbio.1000412) for reporting work involving animal research. Where applicable, authors should refer to any relevant reporting standards documents in this form.

If you have any questions, please consult our Journal Policies and/or contact us: [editorial@elifesciences.org](mailto:editorial@elifesciences.org).

**Sample-size estimation**

- You should state whether an appropriate sample size was computed when the study was being designed
- You should state the statistical method of sample size computation and any required assumptions
- If no explicit power analysis was used, you should describe how you decided what sample (replicate) size (number) to use

Please outline where this information can be found within the submission (e.g., sections or figure legends), or explain why this information doesn’t apply to your submission:

No statistical methods were used to predetermine the sample size. The primary dataset for determining the vicarious pain patterns included data from n = 252 (238 included in the final analysis after quality assessment) healthy subjects acquired in our lab. The number is within the standard range within the field and has been previously validated to be robust against large cross-validation errors (Varoquaux, 2018) and appropriate to facilitate fMRI-based inferences on regions that are most predictive for specific mental processes (Chang et al., 2015). The corresponding information is provided in the introduction section of the manuscript as well as the methods section.

**Replicates**

- You should report how often each experiment was performed
- You should include a definition of biological versus technical replication
- The data obtained should be provided and sufficient information should be provided to indicate the number of independent biological and/or technical replicates
- If you encountered any outliers, you should describe how these were handled
- Criteria for exclusion/inclusion of data should be clearly stated
- High-throughput sequence data should be uploaded before submission, with a private link for reviewers provided (these are available from both GEO and ArrayExpress)

Please outline where this information can be found within the submission (e.g., sections or figure legends), or explain why this information doesn’t apply to your submission:

Repeated cross-validation (10 x 10) was employed to determine the robustness of the findings. Participants with incomplete data, left-handedness or excessive head motion (> 3 mm translation or 3° rotation) were excluded from analyses. No other outliers were excluded. The corresponding procedures are reported in the methods section.

**Statistical reporting**

- Statistical analysis methods should be described and justified
- Raw data should be presented in figures whenever informative to do so (typically when N per group is less than 10)
- For each experiment, you should identify the statistical tests used, exact values of N, definitions of center, methods of multiple test correction, and dispersion and precision measures (e.g., mean, median, SD, SEM, confidence intervals; and, for the major substantive results, a measure of effect size (e.g., Pearson's r, Cohen's d)
- Report exact p-values wherever possible alongside the summary statistics and 95% confidence intervals. These should be reported for all key questions and not only when the p-value is less than 0.05.

Please outline where this information can be found within the submission (e.g., sections or figure legends), or explain why this information doesn’t apply to your submission:

Statistical analysis methods are detailed in the Methods and the Results parts. Statistical tests are reported and justified in the Methods part. Statistical reporting could be found in the Results part as well as the figure legends. We reported p-values along with effect sizes in the results part.

(For large datasets, or papers with a very large number of statistical tests, you may upload a single table file with tests, Ns, etc., with reference to sections in the manuscript.)

**Group allocation**

- Indicate how samples were allocated into experimental groups (in the case of clinical studies, please specify allocation to treatment method); if randomization was used, please also state if restricted randomization was applied
- Indicate if masking was used during group allocation, data collection and/or data analysis

Please outline where this information can be found within the submission (e.g., sections or figure legends), or explain why this information doesn’t apply to your submission:

Participants were not grouped and hence no randomization was performed.

**Additional data files (“source data”)**

- We encourage you to upload relevant additional data files, such as numerical data that are represented as a graph in a figure, or as a summary table
- Where provided, these should be in the most useful format, and they can be uploaded as “Source data” files linked to a main figure or table
- Include model definition files including the full list of parameters used
- Include code used for data analysis (e.g., R, MatLab)
- Avoid stating that data files are “available upon request”

Please indicate the figures or tables for which source data files have been provided:

Statistical and pattern weight images are available on Neurovault (<https://neurovault.org/collections/6332/>). Vicarious pain dataset as well as numerical data and MatLab scripts that were used to generate the figures are available on figshare(<https://figshare.com/articles/Vicarious_pain_dataset/11994498>).
